# Quality Measurement of Consumer Health Questions: Content and Language Perspectives

**DOI:** 10.2196/48257

**Published:** 2024-09-12

**Authors:** Ashwag Alasmari, Lina Zhou

**Affiliations:** 1 Computer Science Department King Khalid University Abha Saudi Arabia; 2 Center for Artificial Intelligence King Khalid University Abha Saudi Arabia; 3 Department of Business Information Systems and Operations Management The University of North Carolina at Charlotte Charlotte, NC United States

**Keywords:** question quality, quality measurement, health questions, information needs, information behavior, information sharing, consumer, health information, health information consumers, quality

## Abstract

**Background:**

Health information consumers increasingly rely on question-and-answer (Q&A) communities to address their health concerns. However, the quality of questions posted significantly impacts the likelihood and relevance of received answers.

**Objective:**

This study aims to improve our understanding of the quality of health questions within web-based Q&A communities.

**Methods:**

We develop a novel framework for defining and measuring question quality within web-based health communities, incorporating content- and language-based variables. This framework leverages k-means clustering and establishes automated metrics to assess overall question quality. To validate our framework, we analyze questions related to kidney disease from expert-curated and community-based Q&A platforms. Expert evaluations confirm the validity of our quality construct, while regression analysis helps identify key variables.

**Results:**

High-quality questions were more likely to include demographic and medical information than lower-quality questions (*P*<.001). In contrast, asking questions at the various stages of disease development was less likely to reflect high-quality questions (*P*<.001). Low-quality questions were generally shorter with lengthier sentences than high-quality questions (*P*<.01).

**Conclusions:**

Our findings empower consumers to formulate more effective health information questions, ultimately leading to better engagement and more valuable insights within web-based Q&A communities. Furthermore, our findings provide valuable insights for platform developers and moderators seeking to enhance the quality of user interactions and foster a more trustworthy and informative environment for health information exchange.

## Introduction

### Background and Motivation

Health information consumers (HICs) are increasingly taking an active role in their health care, turning to a variety of sources for information. Web-based question-and-answer (Q&A) communities are one of these valuable resources, enabling HICs to post and receive answers from fellow community members [1]. Web-based Q&A communities serve as a direct and alternative means of obtaining information compared to search engines, [2].

Articulating health information needs in a concise and understandable question is essential because the relevancy, quality, and nature of the obtained answers are substantially linked to the nature and quality of the question representing the information needs [3,4]. Questions serve as the starting point in the Q&A setting and the primary driver of what might happen next—who responds, and how quality and relevant obtained answers are. However, HICs may experience significant uncertainty in conceiving their information needs and subsequent difficulty in articulating them [5].

Writing high-quality questions can bring many potential benefits. Well-formed questions attract more high-quality answers than poorly formed questions, as subject-matter experts are more likely to assist users who already put in some effort [6]. The quality of the question can significantly impact the probability of receiving helpful answers [7], which eventually drives the popularity of a Q&A community. Previous studies [8-12] show that the features of the questions and the responsiveness to these questions are correlated. More specifically, there are language determinants of the quantity and quality of received answers in web-based communities and social networks [11]. For instance, stating the information needs in a question format, explicitly scoping the audience, and using only 1 sentence leads to high quality and timely answer [11]. Other studies indicate that a high-quality question should provide more details and examples [12]. Thus, HICs need to formulate their information needs in high-quality health questions to receive timely, relevant, and comprehensive answers.

There are 3 streams of research related to question quality. The first one is focused on the characteristics of answers with the assumption that high-quality questions generate answers, and the most straightforward measure of a good-quality question is whether it has received an acceptable answer [13]. In contrast, a poor-quality question likely fails to receive any answers. Some studies have further examined the quality of answers in Q&A communities [14]. This stream of research relies on the outcomes of Q&A interaction, which may not be available. Even if they are available, a lag is expected between the times when an HIC posts a question and when an answer is provided. The second stream of research examines the characteristics of the askers, such as reputation or expertise [15], on the basis that community recognition reflects the ability to construct appropriate and valuable questions. However, the asker’s information is generally not publicly available due to the platform’s privacy policy or the HIC’s privacy concerns. The third stream is on analyzing question characteristics, such as the topics, number of views, and content and language variables that correlate with high-quality answers [16]. Examining question quality based on the question’s intrinsic features holds promise for overcoming the limitations of the first 2 research streams. However, limited research has examined question quality and its measurement, particularly within the context of health-related questions.

This study seeks to enhance our understanding of the intrinsic variables influencing health question quality posed in web-based Q&A communities from the content and language perspectives. Specifically, we address the following primary research question: What are the content and language variables of high-quality questions in web-based health Q&A communities? Answering the question can not only assist HIC in soliciting answers from peer community members and actively engaging themselves in community discussions but also promote the success of web-based Q&A communities.

### Our Proposed Constructs for Health Question Quality

**Figure 1:**
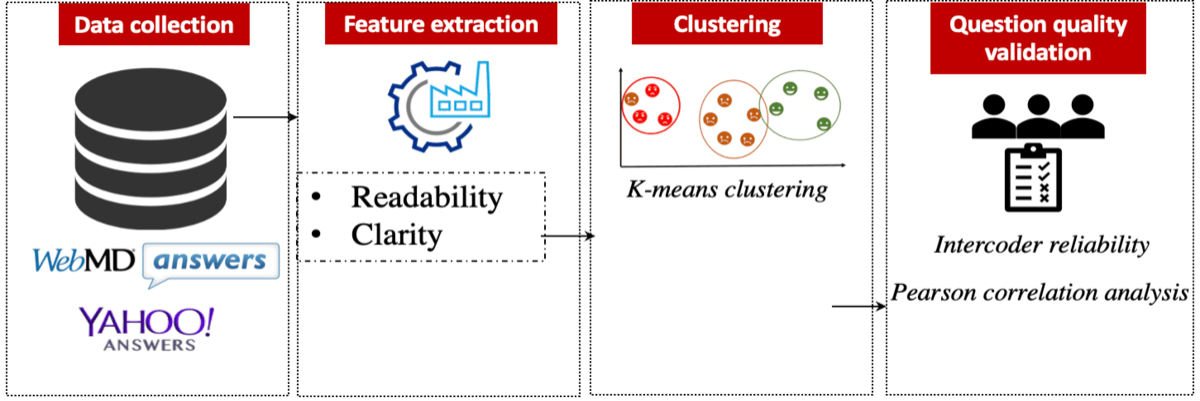
Architecture of health question quality assessment.

In this section, we first introduce our proposed construct of the health question quality. We then develop quantitative measures for the construct using a clustering approach and finally validate the measurements through human assessment. Figure 1 illustrates the architecture of health question quality measurement, including data collection, feature extraction, clustering, and question quality validation.

### Construct Design

We introduce a conceptualization of question quality that centers on 2 key indicators: readability and clarity. We chose these 2 indicators because they represent the variety of measures that impact how people perceive questions. Quality questions should be readable in order to be accessible to a large and diverse audience and be clear about what is expected to be answered. To the best of our knowledge, these indicators have not yet been explored in the context of health-related questions, let alone their collective impacts. These proposed indicators will enable us to uncover fundamental aspects of question quality that would otherwise be missed through solely examining answers. Therefore, our proposed construct will facilitate the timely assessment of question quality before receiving any answer.

Readability relates to how well a question is written, how understandable it is, and the extent to which it is free of unnecessary complexity. The concept of readability is often studied in natural language processing and used as an indicator of article content quality in the digital library [17]. For web-based Q&A communities, we assume that most community members communicate at an average reading level [18]. Accordingly, questions with a high reading level tend to attract fewer potential answers because fewer community members would comprehend the question [18]. Clarity assesses how easily understood a question is and how readily it can be answered. This directly correlates to a higher probability of receiving high-quality answers.

### Construct Measurement

#### Overview

We propose measures for each of the 2 indicators of health question quality construct.

#### Readability

A variety of automated measures, such as the Flesch-Kincaid Grade Level, Gunning Fog Index, SMOG (Simple Measure of Gobbledygook) Index, Coleman-Liau Index, and Automated Readability Index, have been developed to assess text readability [19]. Among them, the Flesch-Kincaid Reading Ease metric (equation 1) is the most widely used [20] and has been adopted in question-answering in various significant contexts [8]. Similarly, we have incorporated it into our study.



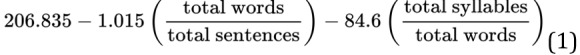



The Flesch-Kincaid readability score uses the average length of sentences and the average number of syllables per word to calculate reading ease. The output is on a scale, which typically ranges from 1 to 100, with lower scores indicating a more difficult text to read. As laypersons typically compose health questions without a medical background, these questions are nonscientific and share similarities with informal discourses encountered in a high-school education setting. Hence, the Flesch-Kincaid Reading Ease method is applicable in this context.

#### Clarity

Clearly expressed questions are more readily understood by other HICs, facilitating relevant and timely answers. For a question to be considered explicit, it should include a minimum of 1 interrogative word [8]. Interrogative words (eg, who, what, where, when, why, and how) can indicate the clarity of health questions. Following the work of Kitzie et al [18], we developed a scale of clarity based on the number of interrogative words normalized by the question length. Specifically, a higher ratio of interrogative words to the total number of words suggests a clearer formulation of the question. We illustrate the measure of clarity with 2 sample questions as the following:

- Q1: “What's the first thing doctors do when a child is diagnosed with chronic kidney disease? And what happens next. Like when you first go in what do they do? What do they tell you? what happens?”
- Q2: “I have been early diagnosed with stage 3 kidney failure. Let me know thoughts on treatments? The condition stems from many years of high blood pressure. I am 55 and hope this doesn't lead to dialysis.”

Question Q1 contains multiple occurrences of interrogative words, such as what and when, accounting for 19.44% of words in the question. They clearly indicate the user’s informational needs. In contrast, question Q2 provides more background information about the HIC’s condition but does not explicitly state his or her question with any interrogative word, which results in a ratio of zero for clarity. Thus, the information needs expressed in Q2 are not as clear as those in Q1. We used linguistic inquiry and word count (LIWC) [21] to extract interrogative words.

#### Overall Question Measurement

One straightforward method to derive an overall quality measure is by averaging the measures of individual indicators, namely readability and clarity. However, assigning equal weight to all indicators may overlook the nuanced differences among individual constructs within questions. In this study, we propose a clustering analysis approach complemented with a human assessment to validate the measurement. This study used the k-means clustering technique [22] to group health questions into a small number of distinct clusters based on question similarities. The parameter k was empirically determined based on the number of clusters’ sets and the proportion of betweenness. We selected the elbow method to determine the number of clusters, which runs the k-means clustering algorithm on the data set for a range of values and then calculate the sum of squared error for each value [22]. Accordingly, we set k to 3. To validate the clustering results, we used the method of human validation.

### Human Validation

Three human judges were recruited to manually assess question quality. Each judge had extensive experience searching for health information on the web and actively participating in web-based Q&A communities. All were English speakers with direct or indirect experience in disease management. We opted not to include human judges with medical backgrounds because web-based Q&A communities tend to attract people without medical expertise, as observed in prior studies [23-25].

We performed stratified sampling by randomly selecting 10% of the questions from each quality cluster. The order of the questions was randomized for each of the human judges. The judges were asked to rate each question independently at 1 of the 3 quality levels: high, average, and low.

The final quality rating of the health questions was determined based on a joint discussion of the 3 judges. Intercoder reliability was assessed with Cohen κ statistics, following the convention of κ>0.70 indicating “substantial” agreement [26]. In addition, we also performed a Pearson correlation analysis between the ratings of the human judges and the results of k-means clustering using the following coding scheme: high=1, average=2, and low=3. The results show that r>0.5, which indicates a very high correlation.

## Methods

### Overview

To investigate the content and language characteristics influencing the quality of health-related questions in web-based Q&A communities, we begin by describing the data set. Subsequently, we introduce content- and language-based variables and highlight the analysis methods used.

### Data Collection

For this study, we collected health questions from Yahoo! Answers, a community-based Q&A site, and WebMD, an expert-based Q&A site. WebMD is one of the most influential web-based health sites, and users were able to post questions for certified health experts to answer in the Q&A section, covering more than 900 health topics. We collected all posts made from August 2008 until the closure of WebMD Answers in 2018. Yahoo! Answers features health as one of the top-level categories. Since its release in December 2005, Yahoo! Answers has become a popular internet reference site worldwide and the most frequented Q&A community in the United States. As of June 2019, the site ranked ninth in global internet traffic and engagement over the past 3 months and seventh in the United States.

Following the prior work [27-30], we treated postings in a question section of a Q&A site as a question regardless of whether they ended with a question mark or not. To investigate question quality in the context of web-based health communities, we focused on kidney disease. This condition, characterized by its prevalence and complex management, provides a valuable case study. Chronic kidney disease (CKD) is a noncommunicable health disease, often accompanied by multimorbidity (frequent co-occurrences with other diseases) [31]. According to the National Kidney Foundation, CKD affects 14% of adults in the United States [32]. In these platforms, patients navigate and manage complex health information, making this study particularly relevant. We screened for queries based on preselected key terms that directly refer to kidney conditions or early signs of CKD [33]. Using an application programming interface, we sampled 400 random questions from Yahoo! Answers and another 400 questions from WebMD relating to the above kidney-related vital terms. In other words, the questions included in our data set must be related to human kidney diseases. We eliminated repeated questions using a combination of user identification and question similarity techniques. In addition, we manually filtered irrelevant questions, including advertisements, those unrelated to kidney-related topics, those not involving human patients, or those associated with student research. For each of the relevant questions, we extracted the title, description, date of question, topic, and number of answers.

### Content-Based Variables

We contextualized the health questions by drawing on relevant findings from a previous study on health information seeking [27]. The 2 main areas of interest in our analyses included health stages of disease development and information shared (ie, demographic and medical information). Further, 2 coders, with medical backgrounds, manually coded the stage of disease development and the type of information shared. To ensure coding consistency, a well-defined coding guideline was followed. Additionally, any discrepancies between the coders’ initial assessments were resolved through decisiveness by a third coder to minimize subjective bias and ensure the accuracy of the coded data. Variables were coded as binary variables (1 indicating presence and 0 indicating absence) of specific information.

- Health stages of disease development: We followed 2 systems in representing the stages of disease development: stages of health questioning [27] and chronic disease stages [34]
  - Stages of health questioning: Managing a disease or condition is an ongoing process, and HIC at different stages often has different levels of information needs [29]. Additionally, HICs may display other information-seeking behaviors based on the nature and extent of their needs. To identify at which stage of their disease HIC asked questions (stage of health questioning), we used a model proposed by Zhang [27] consisting of eight stages: (1) being healthy—at this stage, the questions are related to disease prevention and health promotion, (2) self-diagnosed as being ill, (3) before having a medical test or checkup, (4) after being diagnosed or self-diagnosed as ill, (5) before treatment (such as surgery or medications), (6) during treatment (including medications or exercise); (7) after treatment, and (8) when the disease becomes chronic or reaches the terminal stage. To address data sparsity, we merged stages (2-4) as stage 2 and stages (5-7) as stage 3.
  - Chronic disease stages: We also drew on the stages of chronic illness to understand the questions of HICs who are chronically ill. To this end, we used the Corbin Chronic Illness Trajectory Framework because it includes all stages of chronic disease [34] and is used by clinicians in nursing care and chronic illness management [35]. This trajectory framework is built around the idea that chronic conditions vary and change over time, which consists of nine stages [34]: (1) pretrajectory: before the disease onset; (2) trajectory onset: the appearance of symptoms and diagnosis; (3) stable: condition and symptoms are under control, everyday life is unaffected, illness management is home-centered, and hospitalization not required; (4) unstable: condition and symptoms are not under control, everyday life is disrupted, but care remains centered in the home; (5) acute: symptoms or complications require hospitalization or other measures, everyday life activities are cut back or severely curtailed; (6) crisis: a life-threatening situation that requires emergency care, everyday life is placed on hold; (7) comeback: a return to everyday life activities, possibly with changed ability for everyday life activities; (8) downward: decline associated with increased disability and trouble controlling symptoms, requires adaptation in everyday life activities; and (9) dying: death of the patient. To address data sparsity, we grouped the nine stages into 2: stable and nonstable stages, with the latter covering stages 4 through 9 and the former covering the rest.
- Type of information shared: When HICs communicate their information needs by asking questions, they often include some demographic and medical information related to diagnosis, treatment, and prevention that represent their understanding of their disease [27]. Demographic information includes age, gender, ethnicity, weight, location, and profession. Medical information may pertain to diagnosis, treatment, or prevention. Diagnostic medical information includes symptoms, medical tests, personal and family medical history, etc. Treatment and prevention medical information include treatment options, duration of treatment, lifestyle, prescribed medications, length of hospitalization, etc.

### Language-Based Variables

We leveraged LIWC [21] to extract language-based variables due to the tool’s ability to measure language in multiple dimensions. The value of each dimension is calculated based on the percentage of words related to this particular dimension [21]. For this research, we selected the following variables that can be used to characterize the language and writing style of a health question:

- The total number of words and the number of words per phrase.
- Total number of pronouns: these include personal pronouns and impersonal pronouns.
- Social processes: this includes references to family or friends, as well as biological sex (male and female).
- Time orientation: this includes references to the past, present, and future, which reflect a general time orientation.
- Biological process: this includes references to the body and health.
- Affect: this includes 5-word variables: overall affect, positive and negative emotion, anxiety, anger, and sadness.

### Analysis Methods

A multinomial logistic regression model was used to analyze the content and language variables determining the health question quality. The dependent variable was the overall question quality, and the independent variables were content- and language-based.

### Ethical Considerations

The study's use of health-related questions from a public web-based platform raises potential privacy concerns. To address this, we implemented several measures to protect user privacy. While the data was sourced from a publicly accessible platform, we strictly adhered to the platform’s terms of use (eg, Yahoo’s agreement) for data collection. Due to copyright restrictions, we were unable to directly share the original questions but instead provided unique identifiers. These questions remain publicly accessible with appropriate platform permissions. To further safeguard privacy, all personally identifiable information was carefully removed from the data set. Recognizing the limitations of a small data set focused on a single disease, we implemented measures to mitigate potential biases in data collection and analysis, such as random sampling and rigorous coding guidelines.

## Results

### Descriptive Statistics

Table 1 reports the descriptive statistics and the statistical test for the content-based variables. The table shows that the proportion of medical information included in WebMD questions is significantly higher (*P*<.01) than that in Yahoo! Answers questions. More specifically, medical treatment and medical diagnosis information in WebMD are significantly higher (*P*<.05) than that in Yahoo! Answers. In contrast, demographic information is higher in Yahoo! Answers (*P*<.01) than in WebMD. The comparison results of the stages of general health questioning show that the proportions of all stages except for stage 1 are significantly higher in WebMD (*P*<.01) as compared to Yahoo! Answers. In contrast, the proportions of questions regarding the 3 chronic disease stages are higher in Yahoo! Answers (*P*<.01) than in WebMD.

Table 2 reports the descriptive statistics and the statistical test for language-based variables. It shows that the average word count per question, the total number of pronouns, personal pronouns, and affect in WebMD is significantly lower (*P*<.01) than in Yahoo! Answers. In contrast, the social process is lower in questions from Yahoo! Answers (*P*<.01) than those from WebMD. However, no statistically significant differences were detected in word per sentence and time orientation between the Yahoo! Answers and WebMD platforms (*P*=.96).

Based on the results of k-means clustering, the coders manually analyzed the indicators of the question quality within each of the 3 clusters and provided a quality rating for each cluster separately, ranging from low to high quality. The statistical details of each cluster are provided in Table 3. We discussed the method for determining the number of clusters in the second section (our proposed constructs for health question quality).

- Cluster 1 (high quality) comprised 101 questions with readability ranging between 39.01% and 80.5% and clarity of 33.33% or above.
- Cluster 2 (average quality) had 169 questions with readability ranging between 0.7% and 49.3% and clarity of 12.5% or lower.
- Cluster 3 (low quality) contained 354 questions with readability ranging between 1.5% and 14.7% and clarity being 9.09% or lower. While the clarity range of the current cluster is somehow similar to that of cluster 2, the latter is rated lower than the former on a range of readability.

**Table 1:** Descriptive statistics for content-based variables.

		Yahoo! Answers, n (%)	WebMD, n (%)	*P* value
**Types of information shared**				
	Medical information	147 (46.52)	182 (59.09)	<.001^***^
	Demographic information	100 (31.65)	58 (18.83)	<.001^a^
	Medical diagnosis information	111 (35.13)	154 (50)	<.001^a^
	Medical treatment information	78 (24.68)	96 (31.17)	.035^b^
**Stages of health questioning**				
	1	12 (3.8)	11 (3.57)	.88
	2	55 (17.41)	87 (28.25)	<.001^c^
	3	4 (1.27)	36 (11.69)	<.001^c^
	4	191 (60.44)	64 (20.78)	<.001^c^
	5	54 (17.09)	110 (35.71)	<.001^c^
**Chronic disease stage**				
	1	119 (37.66)	40 (12.99)	<.001^c^
	2	50 (15.82)	18 (5.84)	<.001^c^
	3	147 (46.52)	250 (81.17)	<.001^c^

^a^*P*<.001.

^b^*P*<.05.

^c^P<.01.

**Table 2:** Descriptive statistics for language-based variables.

	WebMD, mean (SD)	Yahoo! Answers, mean (SD)	*P* value
Word count	42.1 (36.9)	60.2 (59.4)	<.001^a^
Word or sentence	13.1 (7.9)	13.1 (7.1)	.96
Total pronouns	12.1 (7.1)	14.3 (7.2)	<.001^a^
Personal pronouns	6.6 (6.3)	7.9 (5.6)	.003^a^
Affect	1.0 (2.5)	1.5 (2.2)	.01^b^
Social process	3.3 (5.1)	6.6 (6.0)	<.001^a^
Time orientation	3.0 (3.8)	2.6 (3.1)	.08

^a^*P*<.001.

^b^*P*<.05.

**Table 3:** Descriptive statistics of quality indicators in the question clusters of different quality levels.

Cluster and indicators		Mean (SD)	Minimum	Maximum
***1 (high quality)***				
	Readability	9.88 (8.95)	39.01	80.5
	Clarity	2.28 (2.35)	9.09	33.33
***2 (average quality)***				
	Readability	9.6 (6.9)	0.7	49.3
	Clarity	1.85 (2.72)	0.0	12.5
***3 (low quality)***				
	Readability	6.67 (2.62)	1.5	14.7
	Clarity	15.35 (5.4)	0	9.09

### Regression Analysis Results

We chose high-quality questions as the reference cluster because it is the expectation of all health questions. Accordingly, the regression coefficients indicate which independent variables significantly discriminate high-quality questions from low- and average-quality questions, respectively.

Tables 4 and 5 provide the coefficient estimates for all outcome comparisons, along with the SE for each independent variable. We found statistically significant differences between the reference quality cluster and the other clusters in some of the stages of health questioning, most types of shared information, and the language variables of the health questions. However, there is no evidence that expressions of anxiety differ among the different quality clusters of the health questions.

Further, 3 of the stages of questioning (before or after diagnosis, when chronically ill, and when in an unstable condition) were strong predictors of high quality among all comparisons with the reference cluster (high-quality questions; *P*<.001; Table 4). This reached statistical significance only when comparing high-quality to low-quality questions for unstable conditions (*b*=0.87, SD 0.34, *P*<.005). Thus, questions relating to diagnosis, or chronic or unstable conditions are less likely to be of high quality. Questions relating to preventative reasons, treatment, and stable stages of chronic disease did not significantly affect the prediction of question quality (*P*>0.1).

Demographic information and diagnostic medical information are significant predictors across the 2 comparisons (*P*<.001). High-quality questions are more likely to include more demographic information than average quality (*b*=–0.84, SD 0.21, *P*<.001) and low quality (*b*=–3.39, SD 1.02, *P*<.001). In addition, high-quality questions are more likely to include more diagnostic medical information than low-quality (*b*=–2.96, SD 0.73, *P*<.001), but least compared to average quality (*b*=1.08, SD 0.19, *P*<.001). Finally, high-quality questions are more likely to include more treatment and prevention information than low-quality questions (*b*=–3.20, SD 1.02, *P*<.001).

Regarding language variables of health questions (Table 5), we found that the number of words (*P*<.001) and the number of words per sentence (*P*<.001) tend to differ significantly between the 2 average and high-quality questions. Longer questions are more likely to be classified as high-quality (*b*=–0.01, SD 0.003, *P*<.001) than low-quality questions. However, high-quality questions are less likely to be associated with a higher word count per sentence than average-quality questions (*b*=0.04, SD 0.01, *P*<.001). That means average-quality questions tend to be overall shorter, but with more words per sentence than high-quality questions.

Regarding the effect and measures of emotions, a statistically significant difference was found between low or average and high-quality questions. High-quality questions were more likely to be effective compared to low-quality (*b*=–2.12, SD 0.03, *P*<.001), and yet less likely to include positive (*b*=2.05, SD 0.04, *P*<.001) or negative (*b*=2.08, SD 0.03, *P*<.001) emotions than low-quality questions (Table 5). The expression of anger was also associated with high-quality questions (*b*=–0.07, SD 0, *P*<.001). In addition, the expression of sadness was associated with high-quality questions (*b*=–0.25, SD 0.06, *P*<.001). However, there is no evidence that expressions of anxiety in health questions differ among the different quality clusters.

Questions that focused on the past were more likely to be high quality compared to low quality (*b*=–0.30, SD 0.14, *P*<.001). In comparison to low-quality questions, high-quality questions were more likely to focus on the future (*b*=–0.16, SD 0.06, *P*<.001). Questions focusing on the present were equally likely to be of any quality.

In addition, we found that among the social processes, only mentions of friends (*b*=–1.74, SD 0, *P*<.001) or male references (*b*=–5.85, SD 0.00, *P*<.001) tend to be strong predictors of high-quality questions compared to low-quality ones. No other differences were detected between any of the other social processes (including family and female references) and other pairs of comparisons (*P*>.05).

**Table 4:** Multinomial logistic regression analysis of the effect of content variables on question quality.^a^

Question and explanatory variable			Average versus high, coefficient (SE)		Low versus high, coefficient (SE)
**Stages of health questioning**					
	Preventative reasons	0.82 (0.51)		–1.24 (0.83)	
	Before or after diagnosis	2.83^b^ (0.78)		0.18 (1.33)	
	Before, after, or during treatment	–0.64 (0.5)		–0.8 (0.69)	
**Chronic disease stages**					
	When chronic	0.54 (0.53)		2.08^b^ (0.68)	
	Stable	–0.41 (0.4)		0.26 (0.52)	
	Unstable	1.16^b^ (0.24)		0.87^c^ (0.34)	
Demographic information			–0.84^b^ (0.21)		–3.39^b^ (1.02)
**Medical information**					
	Diagnostic information	1.08^b^ (0.19)		–2.96^b^ (0.73)	
	Treatment and prevention	0.34^d^ (0.2)		–3.2^b^ (1.02)	

^a^High quality (cluster 1) serving as a reference cluster.

^b^*P*<.001.

^c^*P*<.05.

^d^*P*<.10.

**Table 5:** Multinomial logistic regression analysis of the effect of language variables on question quality.^a^

Explanatory variable	Average vs high, coefficient (SE)	Very low vs high, coefficient (SE)
Word count	–0.01^b^ (0.003)	–0.14^b^ (0.03)
Word or sentence	0.04^b^ (0.01)s	0.22^c^ (0.08)
Total pronouns	–4.30^b^ (0.01)	7.54^b^ (0.01)
Personal pronouns	4.254^b^ (0.01)	–7.53^b^ (0.02)
Impersonal pronouns	4.160^b^ (0.02)	–7.36^b^ (0.02)
Social processes	–0.11^b^ (0.03)	–0.07^c^ (0.03)
Family	0.02 (0.09)	0.00 (0.28)
Friend	–0.29 (0.22)	–1.74^b^ (0)
Female references	0.05 (0.06)	–0.04 (0.25)
Male references	0.11^c^ (0.053)	–5.85^b^ (0)
Past focus	0.11^b^ (0.03)	–0.30^c^ (0.14)
Present focus	0.01 (0.02)	0.02 (0.03)
Future focus	–0.16^c^ (0.06)	–0.06 (0.09)
Body	0.04^d^ (0.02)	0.024 (0.03)
Health	–0.11^b^ (0.02)	–0.06^b^ (0.02)
Affect	0.37 (0.26)	–2.12^b^ (0.03)
Positive emotion	–0.37 (0.26)	2.05^b^ (0.04)
Negative emotion	–0.33 (0.26)	2.08^b^ (0.03)
Anxiety	–0.07 (0.00)	0.12 (0.11)
Anger	0.09 (0.15)	–0.71^b^ (0.00)
Sadness	–0.25^b^ (0.06)	–0.12^d^ (0.07)

^a^High quality (cluster 1) serving as a reference cluster.

^b^*P*<.001.

^c^*P*<.05.

^d^*P*<.10.

## Discussion

### Principal Results

Formulating high-quality questions can potentially bring many benefits, directly impacting the relevancy, quality, and nature of the information acquired. Advancing HICs’ understanding of question content will contribute significantly to identifying quality questions and facilitate HICs in formulating their questions to solicit better answers. This study highlights several content and language variables of high-quality questions. For example, asking questions at various stages of disease development is more likely to be associated with lower-quality questions. On the other hand, high-quality questions are more likely to include demographic and medical information than lower-level quality questions. These results are consistent with a previous study that reported that the chances of a question being answered are higher when background and personal information about the health situation are provided [8]. Health experts will provide relevant and high-quality information when the HIC includes more contextual information about them, such as age, gender, and other background information about the health condition in question.

While high-quality questions conveyed more information using shorter sentences, low-quality questions were shorter overall but contained lengthier sentences. This suggests that clear and concise language expressing specific information needs is crucial for perceived question quality. Complex questions, often with long sentences, may require excessive effort from potential respondents, hindering their willingness to answer [18]. This aligns with our observation that people with more chronic conditions (potentially having more experience formulating clear questions) tend to ask higher-quality questions with shorter sentences.

The expression of positive or negative emotion was more likely to be found in low than high-quality questions, except for anger and sadness which was more likely to be seen in high-quality questions. One possible explanation is that HICs were seeking emotional support in low-quality questions and hence disclosed their emotions more readily [36] than those who posted high-quality questions and were seeking informational help.

Our analysis revealed significant discrepancies in the descriptive statistics between the WebMD Answers and Yahoo! Answers data sets. These differences likely stem from several factors. The platforms themselves cater to distinct audiences. WebMD Answers focused on health information, attracting users with specific medical questions and potentially greater health knowledge. Conversely, Yahoo! Answers served a broader audience with diverse interests, resulting in a wider range of question complexity and potentially lower health expertise. Despite these disparities, we believe that merging the data sets offers valuable insights. The combined data set encompasses a broader range of health-related questions and user experiences. This allows us to explore a more comprehensive picture of health information-seeking behavior, potentially revealing patterns not evident within a single platform.

### Research Contribution

One of the main contributions of this study lies in quantifying health question quality. Although other quality measures already exist, such as receiving satisfactory answers and subjective judgment of quality [9], improving the interaction between users in health-related Q&A communities is of paramount importance. Any further insight into improving the presentation and expression of HIC needs is welcomed.

Unlike previous studies that focus on either the answers or the askers’ profiles to measure the question quality in web-based Q&A communities, this study defined question quality as objective measures of the questions themselves based on their readability and clarity. More specifically, this research explores the idea that questions may share similar quality features. Rather than scoring each question on predefined measures, it used k-means clustering to facilitate the analysis and divided question quality into 3 clusters. Based on the means of each question quality measure, we identified high, average, and low-quality clusters. The results of the clustering process and the subsequent validation with human assessment support the idea that grouping questions is a viable analysis method because of shared characteristics.

Further, 1 final contribution of this study is that the proposed framework for measuring question quality is independent of the application context. The 2 measures, readability and clarity can be applied or extended to other types of web-based Q&A communities beyond health care.

### Research Implications

This new question of quality measurement has significant research implications. First, compared to alternative quality measures (eg, receiving satisfactory answers or based on the asker’s profiles [8,13]), the textual features of questions can provide a timely assessment of quality based on the characteristics of the questions themselves without relying on answers. This is particularly important for researchers aiming to build models that automatically predict or evaluate the quality of large volumes of health questions in web-based Q&A communities. Second, this study establishes a foundation for developing a set of objective quality criteria that can be used to create classifiers for identifying high-quality questions in the future. Such work will require the identification of those content and language variables most relevant to the specific health subject.

This study also offers practical suggestions for improving the effectiveness of web-based Q&A communities. The findings of this study may guide designers and developers of Q&A systems to design community support systems that encourage user contributions and control quality. Based on the measurement methods of question quality introduced in this work, Q&A communities could develop automated systems for prescreening the quality of health questions before HICs submit them to the community and facilitate the asker in formulating high-quality questions. Such approaches could include using an iterative feedback system, query expansion, or syntax checking correction. Quality questions are likely to lead to quality answers, critical to user engagement in those communities.

Iterative feedback mechanisms, such as query reformulation in interactive information retrieval [37], have demonstrated their effectiveness in helping users refine information needs based on initial relevance judgments and subsequent system feedback. Within the Q&A context, archived answers to similar questions could be presented to the asker, and the asker could pick the questions that are most relevant to their question to check out their answers. The asker may not only learn from others how to write questions but also find answers to their questions directly. In addition, by understanding the textual features of high-quality questions, consumers can tailor their questions to elicit optimal responses. Our research provides consumers with concrete steps to improve their questions. Highlighting details such as demographics, medical history, and symptoms clearly and concisely can lead to more targeted and relevant answers. This empowers consumers to make informed health care decisions based on reliable information.

One long-term implication of this study lies in helping health care professionals or artificial intelligence systems improve their performance by adding a layer of quality assurance before the question is processed. Already, artificial intelligence is finding its uses in health care such as diagnosis, personalized care, and treatment [38], and it relies on quality data to perform well. In addition, health care providers will also benefit from receiving high-quality questions that contain all relevant information at the outset when providing web-based health advice or telemedicine due to their limited availability. This is particularly important because health professionals rely more on the patient’s information than the examination findings when managing a patient [39].

### Limitations

One of the main limitations is the small number of health questions used in our study. The increasing diversity of analyzed health questions will strengthen the generalization of our conclusion. Future studies may include more health questions and use an automated approach to analyze the content variables of health questions. In addition to not fully exploring platform effects, we examined health questions from only 2 distinct types of web-based Q&A communities. Health questions are posted on diverse web-based platforms with varying features and functionalities. This limited sample size and lack of platform-specific analysis restrict the generalizability of our findings to other types of Q&A communities. Future research could expand the scope by including a broader range of Q&A platforms and investigating how platform-specific factors such as guidelines, moderation, and user demographics interact with question characteristics to influence response dynamics across diverse web-based communities. This comprehensive approach would provide a deeper understanding of how the web-based environment shapes user behavior and information exchange in different Q&A contexts. Furthermore, this study establishes a strong foundation for investigating question quality assessment in the context of kidney disease. Future research can then expand on this work by conducting empirical validations in other chronic diseases.

In addition, the validation of this new quality measurement was based on human judges who have experience in finding web-based information but not necessarily in answering other HIC questions. Ideally, further validation with other quality measures that have already been evaluated will add another layer of validity to this study. For example, answers can reveal additional insights about the quality of the questions, and future exploration of textual quality indicators should also consider the quality of the answers to the questions. Our new quality measures could potentially be used to analyze the characteristics of high-profile HIC queries in web-based forums. While our current focus is on measures such as readability and clarity, future research can explore prompt engineering, showing how users craft effective questions for health care information systems. The rise of platforms such as ChatGPT highlights this need, as our findings provide a foundation for equipping users with the skills to formulate high-quality prompts regardless of the platform. Longitudinal studies can then assess the real-world impact on information access and communication success in AI-powered health care.

### Conclusions

The increasing popularity and usage of web-based Q&A communities for seeking health information calls for an investigation into factors influencing content quality and ways to improve the quality of questions. By identifying the content and language variables of health questions that affect the quality, this research contributes to a deeper understanding of how users can formulate effective questions that receive accurate and relevant responses. This research would offer meaningful suggestions for platforms’ managers and users as well. This knowledge can empower consumers to become more active participants in their web-based health information–seeking journeys. By formulating concise, specific, and focused questions, people can maximize the effectiveness of web-based Q&A platforms and increase the likelihood of receiving high-quality answers from health care professionals and other informed users. Furthermore, our findings provide valuable insights for platform managers seeking to enhance the quality of user interactions within their communities. By promoting best practices for question formulation through educational resources and user guidance, web-based Q&A platforms can foster a more efficient and trustworthy environment for the exchange of health information. This research paves the way for future investigations into the dynamic interplay between user behavior, platform characteristics, and response quality within web-based health communities. Through continued exploration, we can work toward optimizing the web-based environment for effective health information access and use.

## Abbreviations

CKD: chronic kidney disease
HIC: health information consumer
LIWC: linguistic inquiry and word count
Q&A: question-and-answer
SMOG: Simple Measure of Gobbledygook

## References

[1] Shah C, Oh S, Oh JS (2009). Research agenda for social Q&A. Library & Information Science Research.

[2] Liu Q, Agichtein E, Dror G, Maarek Y, Szpektor I (2012). When web search fails, searchers become askers.

[3] Barry CL (1994). User-defined relevance criteria: an exploratory study. J Am Soc Inf Sci.

[4] Taylor RS (1968). Question-negotiation and information seeking in libraries. CRL.

[5] Belkin NJ (1980). Anomalous states of knowledge as a basis for information retrieval. Can J Inf Libr Sci.

[6] Ameri F, Keeling K, Salehnejad R (2020). You get what you pay for on health care question and answer platforms: nonparticipant observational study. J Med Internet Res.

[7] Baltadzhieva A, Chrupa\la G (2015). Question quality in community question answering forums: a survey. ACM SIGKDD Explor Newsl.

[8] Shah C, Radford ML, Connaway LS, Choi E, Kitzie V (2013). "How much change do you get from 40$?"- Analyzing and addressing failed questions on social Q&A. Proc of Assoc for Info.

[9] Treude C, Barzilay O, Storey MA (2011). How do programmers ask and answer questions on the web?.

[10] Li B, Jin T, Lyu M, King I, Mak B (2012). Analyzing and predicting question quality in community question answering services.

[11] Teevan J, Morris M, Panovich K (2021). Factors affecting response quantity, quality, and speed for questions asked via social network status messages. http://www.aaai.org/ocs/index.php/ICWSM/ICWSM11/paper/download/2795/3219.

[12] Nasehi SM, Sillito J, Maurer F, Burns C (2012). What makes a good code example?: A study of programming Q&A in StackOverflow.

[13] Chatterjee P, Damevski K, Kraft NA, Pollock L (2021). Automatically identifying the quality of developer chats for post hoc use. ACM Trans Softw Eng Methodol.

[14] Fu H, Oh S (2019). Quality assessment of answers with user-identified criteria and data-driven features in social Q&A. Information Processing & Management.

[15] Shi J, Du W, Xu W (2018). Identifying Impact Factors of Question Quality in Online Health Q&A Communities: an Empirical Analysis on MedHelp. PACIS 2018 Proceedings.

[16] Zhao Y, Wu L, Zhang J, Le T (2021). How question characteristics impact answer outcomes on social question-and-answer websites. J Glob Inf Manag.

[17] Rassbach L, Pincock T, Mingus B (2007). Exploring the feasibility of automatically rating online article quality. Int Wikimedia Conf.

[18] Kitzie V, Choi E, Shah C (2013). Analyzing question quality through intersubjectivity: world views and objective assessments of questions on social question-answering. Proc Am Soc Info Sci Tech.

[19] Zhou S, Jeong H, Green PA (2017). How consistent are the best-known readability equations in estimating the readability of design standards?. IEEE Trans Prof Commun.

[20] Kincaid J, Fishburne R, Rogers R, Chissom B (1975). Derivation of new readability formulas (Automated Readability Index, Fog Count And Flesch Reading Ease Formula) for navy enlisted personnel. Institute for Simulation and Training.

[21] Pennebaker JW, Chung CK, Ireland M, Gonzales A, Booth RJ (2007). The development and psychometric properties of LIWC2007. LIWC Inc, Austin, Texas 78703 USA conjunction with LIWC2007 Softw Progr LIWC Inc, Austin, Texas 78703 USA in Conjunction with the LIWC2007 Software Program.

[22] MacQueen J (1967). Some methods for classification and analysis of multivariate observations. Proc 5th Berkeley Symp Math Stat Probab.

[23] Tavakoli L, Zamani H, Scholer F, Croft WB, Sanderson M (2022). Analyzing clarification in asynchronous information-seeking conversations. J Assoc Inf Sci Technol.

[24] Liu Y, Bian J, Agichtein E (2008). Predicting information seeker satisfaction in community question answering.

[25] Rath M, Shah C, Floegel D (2017). Identifying the reasons contributing to question deletion in educational Q&A. Proc. Assoc. Info. Sci. Tech.

[26] Landis JR, Koch GG (1977). The measurement of observer agreement for categorical data. Biometrics.

[27] Zhang Y (2010). Contextualizing consumer health information searching: an analysis of questions in a social Q&A community.

[28] Roberts K, Demner-Fushman D (2016). Interactive use of online health resources: a comparison of consumer and professional questions. J Am Med Inf Assoc.

[29] Alasmari A, Zhou L (2021). Share to seek: the effects of disease complexity on health information—seeking behavior. J Med Internet Res.

[30] Alasmari A, Zhou L (2019). How multimorbid health information consumers interact in an online community Q&A platform. Int J Med Inform.

[31] Coyne DW (2011). CKD Medscape C Expert Column Series: Issue 3 - Management of Chronic Kidney Disease Comorbidities. Medscape.

[32] (2021). Centers for Disease Control and Prevention.

[33] (2019). Kidney problem symptoms, causes, and types. American Kidney Fund.

[34] Corbin JM (1998). The Corbin and Strauss Chronic Illness Trajectory model: an update. Sch Inq Nurs Pract.

[35] Hinkle JL, Cheever KH (2018). Brunner & Suddarth's Textbook of Medical-Surgical Nursing.

[36] Wang YC, Kraut RE, Levine JM (2015). Eliciting and receiving online support: using computer-aided content analysis to examine the dynamics of online social support. J Med Internet Res.

[37] Hersh W, Shortliffe EH, Cimino JJ (2021). Information retrieval. Biomed Informatics Comput Appl Heal Care Biomed.

[38] Horgan D, Romao M, Morré SA, Kalra D (2019). Artificial intelligence: power for civilisation—and for better healthcare. Public Health Genomics.

[39] Kuziemsky C, Maeder AJ, John O, Gogia SB, Basu A, Meher S, Ito M (2019). Role of artificial intelligence within the telehealth domain. Yearb Med Inform.

